# Supplementary material for: UV-Activated Au Modified TiO2/In2O3 Hollow Nanospheres for Formaldehyde Detection at Room Temperature
Source: Materials (Basel). 2023 May 26;16(11):4010. doi: 10.3390/ma16114010 (PMC10254182; doi:10.3390/ma16114010)
Supplement: Supplementary file 1 [file materials-16-04010-s001.zip › materials-2390980-supplementary.pdf]

materials

MDPI

Supplementary Materials

UV-Activated Au Modified TiO_2/In_2O_3 Hollow Nanospheres for Formaldehyde Detection at Room Temperature

Su Zhang [1], Baoyu Huang [1], Zenghao Jiang [1], Junfan Qian [1], Jiawei Cao [1], Qiuxia Feng [2], Jianwei Zhang [3,*] and Xiaogan Li [1,4]

[1] School of Microelectronics, Dalian University of Technology, Dalian 116024, China; postsuz@163.com (S.Z.); huangby@dlut.edu.cn (B.H.); mistyayaya@163.com (Z.J.); junfanqian@126.com (J.Q.); c_jw2000@163.com (J.C.); lixg@dlut.edu.cn (X.L.)
[2] School of Information and Control Engineering, Qingdao University of Technology, Qingdao 266520, China; fengqiuxia@qut.edu.cn
[3] School of Artificial Intelligence, Dalian University of Technology, Dalian 116024, China
[4] Key Laboratory of Integrated Circuit and Biomedical Electronic System, Dalian University of Technology, Dalian 116023, China
* Correspondence: jwzhang@dlut.edu.cn

The target gas vapors were obtained from the corresponding liquids. Liquid organics such as ethanol, toluene, and acetone are injected through a microinjector into the evaporator in the testing chamber to produce the target gases. The concentration of the target gas is calculated by the following formula:

$$C = (22.4 \times \rho \times d \times V_1)/(M \times V_2) \quad \text{(S1)}$$

where C (ppm) is the target gas concentration; ρ (g/mL) is the density of the liquid; d is the purity of the liquid; V_1 (μL) and V_2 (L) are the volumes of the liquid and the test chamber, respectively. M (g/mol) is the molecular weight of the liquid. For pure solute solutions such as acetone, absolute ethanol, and toluene (99.9 wt%), the corresponding pure target gas can be obtained by volatilization of the solution directly. However, for solutions of non-pure solutes such as formaldehyde (40 wt%) and ammonia (35 wt%), the influence of solvent (water) volatilization on the gas sensing test needs to be considered.

In order to exclude the effect of water molecules in the formaldehyde solution and ammonia on the gas response, we took formaldehyde solution as an example. Table S1 shows the composition of the aqueous formaldehyde solutions used for the preparation of the formaldehyde vapors.

Table S1. Composition of the aqueous formaldehyde solutions used for the preparation of the formaldehyde vapors.

Targeted HCHO (ppm)	2	5	10	20
Total volume of HCHO solution (μL)	0.33	0.82	1.65	3.3
Volume of the corresponding H_2O (μL)	0.2	0.5	0.99	1.98
Concentrations of the corresponding H_2O (ppm)	4.93	12.3	24.7	49.3

In this work, when the testing formaldehyde concentration was 10 ppm, 24.7 ppm of water molecules will be introduced. As shown in Figure S1, the accompanying water produced by the solution had almost no effect on the $Au/TiO_2/In_2O_3$-based sensor. After contact with the water vapor, the signal of the sensor hardly changed. Therefore, this work will exclude the effects of the water in the formaldehyde solution and ammonia on the gas sensing properties. During the process of recovering to the background, the front window of the chamber is opened, and the water vapor and the target gas in the chamber is diffused spontaneously.

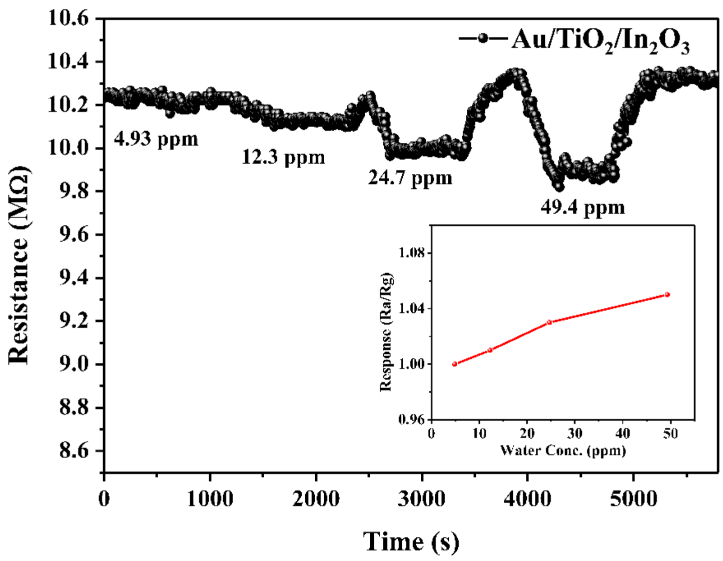

Figure S1. Response curves of the Au/TiO_2/In_2O_3 nanocomposite-based sensor under UV activation to different concentrations of water vapor at room temperature.
